# The Spectrum of Anti-Chromatin/Nucleosome Autoantibodies: Independent and Interdependent Biomarkers of Disease

**DOI:** 10.1155/2014/368274

**Published:** 2014-04-03

**Authors:** Sonal Mehra, Marvin J. Fritzler

**Affiliations:** ^1^Department of Clinical Immunology, JIPMER, 605006 Puducherry, India; ^2^Faculty of Medicine, University of Calgary, 3330 Hospital Drive NW, Calgary, AlB, Canada T2N 4N1

## Abstract

Autoantibodies directed to chromatin components date back to the discovery of the LE cell and the LE cell phenomenon circa 1950, and subsequent evidence that major components of that reaction were chromatin components and histones in particular. Over time, immunoassays ranging from ELISA and line immunoassays to more modern bead-based assays incorporated histone and DNA mixtures, purified histones, and purified nucleosomes leading to a more thorough understanding of the genesis and pathogenetic relationships of antibodies to chromatin components in systemic lupus erythematosus and other autoimmune conditions. More recently, interest has focussed on other components of chromatin such as high mobility group (HMG) proteins both as targets of B cell responses and pro-inflammatory mediators. This review will focus on immunoassays that utilize chromatin components, their clinical relationships, and newer evidence implicating HMG proteins and DNA neutrophil extracellular traps (NETs) as important players in systemic autoimmune rheumatic diseases.

## 1. Introduction

Eukaryotic chromatin is comprised of approximately 40% DNA, 40% histones, 20% nonhistone proteins (i.e., HMG proteins), RNA, and other macromolecules. The fundamental subunit of chromatin is the mononucleosome, which is composed of ~180 base pairs of DNA and two molecules each of the core histones H2A, H2B, H3, and H4 and one of the linker histone H1. The core histones are organized as a histone octamer (containing two H2A-H2B dimers and one H3-H4 tetramer) around which 146 base pairs of DNA are wrapped, thus constituting the “core particle.” This structure is stabilized by histone H1 which binds across the surface of the nucleosome [1]. The periodic arrangement of nucleosomes along DNA strands gives chromatin a “beads on a string” appearance in electron micrographs [2]. The “beads” representing mononucleosomes can be isolated by digesting the internucleosomal linker DNA with micrococcal nuclease (reviewed in [3, 4]).

Human autoantibodies that bind to chromatin targets can be divided into those that recognize dsDNA, protein components of chromatin (i.e., histones, HMG proteins), mononucleosomes, or macromolecular components of nucleosomes as represented by low salt extracted nucleosomes (core particle) [3, 5–7]. Schematically, the family of antinucleosome autoantibodies (ANuA) are primarily directed against histone epitopes localized primarily to exposed domains of native chromatin (i.e., carboxyl terminal tails of core histones), double-stranded DNA (dsDNA), and conformational epitopes created by the interaction between dsDNA and core histones (reviewed in [3, 8]). This review discusses recent studies that explored the pathogenicity, diagnostic relevance, and clinical impact of anti-dsDNA and ANuA with a primary focus on SLE and an overview of more recent advances that are impacting on this field of study and clinical applications.

## 2. Anti-dsDNA Antibodies

Anti-dsDNA are quite specific for SLE, although they have been found in normal individuals where they are mostly the IgM isotype as encoded by germline DNA with few or no somatic mutations [9, 10]. These IgM belong to a family of natural autoantibodies, tend to have low affinity and avidity binding characteristics, and display polyreactivity [11]. For the most part, they are not pathogenic [12], demonstrate geographical differences in frequency [13], and may be protective by virtue of possessing enzymatic activity (abzymes) that can degrade nucleic acids [11]. By comparison, pathogenic anti-dsDNA antibodies are thought to be high-avidity IgG isotypes that react with dsDNA and are somatically mutated as expression of an antigen driven selection process [14, 15]. The natural anti-dsDNA antibodies are produced by a B1 (CD5+) B cell subpopulation, while the pathogenic subsets are secreted by B2 (CD5−) B lymphocytes [16]. The naive B cells specific for ssDNA may clonally expand if stimulated by immunogenic DNA and gain specificity for dsDNA as a consequence of somatic mutations under antigenic stimulation pressure [15].

Autoantibodies to dsDNA were first recognized as an important serological marker for the diagnosis of idiopathic SLE, and eventually both the American College of Rheumatology and Systemic Lupus International Cooperating Clinics (SLICC) criteria for classification of the disease included the presence of these autoantibodies as a formal criterion [17, 18]. Antibodies directed against dsDNA and nucleosomal chromatin have been reported as sensitive biomarkers for the diagnosis of SLE and quantitatively associated with disease activity [8, 19]. Historically, anti-dsDNA autoantibodies in particular were associated with renal involvement [20–23] and they have also been found in immune complex deposits in the glomeruli of SLE patients [24]. Depending on the diagnostic platform used for their detection, anti-dsDNA antibodies are found in approximately 50% of SLE patients [3, 24]. Besides anti-dsDNA, nucleosome-specific antibodies and nucleosome-antinucleosome immune complexes have also been shown to play a major role in the pathophysiology of SLE [23, 25].

## 3. Anti-Nucleosome Antibodies (ANuA)

By comparison, ANuA are a more sensitive biomarker of SLE than anti-dsDNA and are almost exclusively found in SLE and in much lower frequency in systemic sclerosis (SSc), mixed connective tissue disease, and other systemic autoimmune rheumatic diseases (SARD) [26]. Several published studies evaluated ANuA in SLE with various findings [27–30]. The point prevalence of ANuA in SLE varies from 50% to 90% [31, 32] and their presence can be used, in conjunction with clinical findings and other laboratory tests, to support the diagnosis of SLE and certain cases of drug induced lupus (DIL) [3]. Recently, newer immunoassay platforms, including multiplexed microarrays used for detecting ANuA and anti-dsDNA, have been found to be promising in assessing disease activity, especially when anti-dsDNA antibodies are negative [27].

## 4. Nucleosomes Drive Anti-Chromatin Autoantibody Production 

SLE is characterized by the production of both antigen driven autoantibodies such as anti-dsDNA and anti-histone antibodies and polyclonal, apparently nonspecific, autoantibodies. Precise mechanisms leading to production of these autoantibodies are still unclear, but several data suggest that the nucleosome plays a key role [23–25]. Since purified DNA has been known to be a poor immunogen [33, 34] and the immune response is most commonly directed to ribo- and deoxyribonucleoproteins (i.e., small nuclear and small cytoplasmic RNPs such as Sm, U1RNP, SS-A/Ro60), it seems more plausible that the nucleosome is the primary antigen that drives the anti-dsDNA [9] and anti-histone responses via inter- and intramolecular epitope spreading.

An understanding of the sources of the nucleosomes that drive the anti-chromatin response is becoming more and more complex. It has been held for almost two decades that aberrant apoptosis and a reduced clearance of apoptotic cells by phagocytes may lead to an increased exposure of apoptotic nucleosomes to the immune system (reviewed in [31, 35, 36]). It should be added that although a lot of attention has focussed on apoptosis as a key paradigm in autoantigen release and the resulting B and T cell responses, other mechanisms of autoantigen release from necrotic or damaged cells [37–39], circulating microparticles [40, 41], or DNA neutrophil extracellular traps (NETs) [39], discussed later in this review, need also to be taken into consideration.

Nevertheless, a prevailing paradigm is that nucleosomal constituents, which have been cleaved and modified (i.e., acetylated, methylated) during the process of apoptosis [42, 43], escape normal clearance and express epitopes that are not normally encountered by the immune system [36]. This may lead to breaking of tolerance via polynucleosomes produced during cell apoptosis and their subsequent binding to activated monocytes which may present these antigens cells to CD4+ T-helper cells, inducing an antigen-driven response [44]. In murine lupus, ANuA antedate many other autoantibodies [45, 46] and nucleosome-specific CD4+ T cells are detected earlier than the pathogenic autoantibodies, suggesting that cellular immunity may play a key role in triggering and the onset of disease [45, 47]. Lartigue et al. [48] studied the* lpr* mutation in murine lupus and found that the Toll-like receptor 9 (TLR9) is absolutely required for the ANuA response but not for anti-dsDNA antibody production. As a general paradigm, apoptosis results in modified chromatin components appearing at the surface of apoptotic cells, the removal of apoptotic debris is defective, and the massive release of these nucleosomes into the circulation incites their recognition by the immune system (T and B cells) and the production of ANuA [49].

A number of reports have discussed the role of circulating microparticles generated* in vitro* that also displayed DNA and other nucleosomal structures in an antigenic form [40, 41]. These microparticles may be related to blebs that form during cell death and contain both cytoplasmic and nuclear components such as DNA and RNA [50]. It was suggested that the blood of SLE patients can contain microparticles with bound IgG favouring a type of immune complex that may contribute to pathogenesis of SLE [40].

The notion that one of the primary B cell targets in SLE is the nucleosome is supported by a number of observations. Autoantibodies directed against histones are found in 95–100% of DIL sera and also in 70% of SLE [3, 51]. Early studies suggested that subunits of the nucleosome rather than free histones are highly antigenic in procainamide-induced lupus [52, 53]. Subsequent studies demonstrated that antibodies directed against the (H2A-H2B)-DNA subnucleosome particle were a serological feature of DIL [4, 54]. These studies implicated the nucleosome rather than its component proteins or DNA as the immunogenic stimulus for antibody appearance in drug-induced autoimmunity (DIA) or in DIL in the absence of symptoms (reviewed in [3, 4]). This was supported by observations that ANuA can be detected very early during the disease (i.e., before intramolecular epitope spreading to anti-dsDNA and antihistone responses) and is potentially nephritogenic in lupus mice [55, 56]. Thus, breaking peripheral tolerance leading to ANuA, immune complex formation and activation of complement could be related to nucleosomes rendered immunogenic by being present in large excess and/or harboring modifications. It has been shown that a large part of ANuA activity is attributable to nucleosome-restricted autoantibodies and is distinct from anti-dsDNA and anti-histone reactivity [56, 57]. In addition, it has been demonstrated that antigens other than DNA can initiate the anti-dsDNA antibody responses [58, 59]. Furthermore, there is evidence that IgG anti-dsDNA autoantibodies can shuttle nucleic acid fragments through the plasma membrane causing activation and secretion of inflammatory cytokines contributing to the pathogenesis in SLE (reviewed in [60]). The debate about autoantibodies penetrating living cells has persisted for almost four decades [61] and despite resistance to the idea [62], it is a concept that refuses to go away (reviewed in [63]).

## 5. Diagnostic Assays for the Detection of Anti-dsDNA and ANuA

Over the last five decades since their first description, several methods for anti-dsDNA detection have been developed (reviewed in [64, 65]). The most commonly used immunoassays are the* Crithidia luciliae* immunofluorescence test (CLIFT) and various enzyme-linked immunoassays (ELISA). More recently, other methods include the use of strips blotted with the DNA molecules (immunoblotting (IB) and line immunoassays (LIA)) and the use of microarrays and addressable laser bead immunoassays (ALBIA) [66–68]. There is evidence that the various immunoassays differ in sensitivity and/or specificity and may identify different autoantibodies with different diagnostic and prognostic values. Although the Farr radioimmunoassay was widely acclaimed as the assay of choice because the results can be correlated with global SLE activity and renal and vasculitis involvement, it has been largely replaced by nonradioisotopic techniques, mainly indirect immunofluorescence tests using purified circular dsDNA as would be represented in the* Crithidia luciliae* kinetoplast and purified dsDNA in an ELISA [64, 65].

In a multicenter study of four different anti-dsDNA immunoassays, autoantibody titres detected by EliA and FARR assay were correlated with SLE disease activity [69]. It has been shown that low-avidity autoantibodies are primarily detected by ELISAs (as well as complement fixation, hemagglutination, and polyethylene glycol (PEG) assays), medium-to-low-avidity autoantibodies by CLIFT, while high-avidity antibodies are most reliably detected by the Farr assay [15]. ALBIA has also been developed to detect anti-dsDNA but this assay platform has been troubled with lack of precision and a challenge in correlating the results with other established platform assays [70–72].

A more recently developed high avidity anti-dsDNA IgG ELISA is reported to have highly specific performance characteristics for the SLE although it is less sensitive than certain other dsDNA IgG assays [73]. In a multicentre study of SLE patients, anti-dsDNA antibodies were detected in serum by means of a “Farrzyme” assay, fluoroenzyme immunoassay (EliA), CLIFT, or Farr [74]. The sensitivity for SLE ranged from 66% with Farrzyme to 95% with Farr, with about 90% specificity for all the methods tested. The four methods correlated with disease activity and renal or haematologic involvement and showed a negative association with central nervous system disease [74]. Another study suggested that these EIA tests may replace CLIFT as a screening test and the Farr assay as a specific test for anti-dsDNA antibody detection [75]. Therefore, screening with the sensitive ELISA detects most anti-dsDNA antibodies irrespective of pathogenic impact [76], and follow-up positive ELISA results by more stringent assays (CLIFT, FARR assay with circular dsDNA as antigen, EliA anti-dsDNA assays, or solution-phase ELISA) will determine the presence of potentially more pathogenic anti-dsDNA antibodies [77, 78].

ELISA techniques for ANuA detection differ in terms of the antigen preparations used. Some methods use purified nucleosomal particles obtained by reconstituting histone core proteins or histone dimers onto DNA; others use purified chromatin generally obtained by digestion with micrococcal nuclease and subsequent removal of histone H1 and HMG proteins with 0.5 M NaCl at a neutral pH [79]. Because it was demonstrated that the presence of the H1 histone in the preparation of nucleosomes was a cause of “false positive reactions,” many manufacturers modified the antigenic preparations by stripping the H1 histone and, in the course of that treatment, also most HMG proteins from the nucleosomal complex [35]. Hence, assays that employ reconstituted nucleosomes or nucleosomes stripped of H1 do not measure HMG antibodies as well. Studies of SLE sera using assays that employed H1-stripped nucleosomes and those that used whole nucleosomes were shown to have comparable sensitivity (61% versus 59%), but the specificity of H1-stripped nucleosome was much better than that for assays that used whole nucleosomes (95.7% versus 87.5%) (reviewed in [8]).

Bardin et al. [80] found that a multiplexed addressable laser bead immunoassay (BioPlex 2200: BioRad) used for the simultaneous detection of both ANuA and anti-dsDNA autoantibodies increased the sensitivity for SLE from 68–70% when only one antibody was detected (anti-dsDNA or ANuA) to 78% when both antibodies were detected. This was suggested to be especially useful in followup of SLE patients with active lupus nephritis. Similarly, in a retrospective study of 764 patients with rheumatic diseases, Bose et al. used the multiplexed Bioplex 2200 ANA screen to simultaneous determine of autoantibodies to extractable nuclear antigens and ANuA [81]. The sensitivity, specificity, positive predictive value, and negative predictive value of the ANuAs in SLE were 62.4%, 91.5%, 50.4%, and 94.6%, respectively. Of note, no correlation was found between the ANuA and lupus glomerulonephritis or anti-dsDNA antibodies. In our experience it is very difficult to multiplex assays where ligands or components of macromolecular complexes (i.e., nucleosomes, DNA, histones) are included in the same assay. Therefore, although newer advancements like microarrays and multiplexed immunoassays have great potential for research and diagnostic applications in SLE and other SARD [66], a number of technical hurdles need to be overcome.

## 6. Clinical Associations of Anti-dsDNA and ANuA in SLE

At the outset, it must be made clear that ANuA represents a very complex autoantibody system comprising a variety of potential protein targets, epitopes, and B cell responses leading some to take a rather nihilist view of their clinical value [82]. Nevertheless, extensive literature has consistently shown that ANuA do have meaningful clinical correlates. ANuA and anti-dsDNA autoantibodies have been associated with lupus disease activity and higher SLEDAI scores [4, 26, 80, 81, 83–89] (see Table 1). The reported prevalence of ANuA in SLE ranged from 50% to 100% [57, 90, 91] and the corresponding specificity between 90% and 99% [26–29, 57, 85, 86, 89, 90, 92–94]. A review of ANuA and anti-dsDNA autoantibodies showed greater diagnostic sensitivity for ANuA (59.9%) than for anti-dsDNA (52.4%), with a comparable specificity (94.9% versus 94.2%, resp.) (reviewed in [8]). The difference between the various studies is attributed to the makeup of the clinical cohorts but especially to technical issues such as antigen purification and the adopted cutoff. In a study by Kim et al. [95], the sensitivity of ANuA in SLE was 98.8% and the specificity was 78.3%. By comparison, Su et al. [96] reported that the sensitivity and specificity of ANuA in SLE were 61.8% and 97.6%, respectively, and Simón et al. [85] reported that ANuA had a sensitivity of 100% and specificity of 97% for SLE diagnosis. In a South African study, Tikly et al. [97] reported that the sensitivity, specificity, positive predictive value, and negative predictive value of ANuA were 45.3%, 94.3%, 88.6%, and 63.6%, respectively. In this study, the presence of ANuA was strongly associated with anti-dsDNA antibodies (OR = 3.4, *P* < 0.0005) and anti-histone antibodies (OR = 15.7, *P* < 0.00001). Ghillani-Dalbin et al. [98] studied 1696 patients with various autoimmune diseases and reported that 78% of SLE were positive for ANuA while 43% in the SLE group were positive for ANuA and negative for anti-ds-DNA antibodies, indicating that ANuA is an independent biomarker in SLE. Cairns et al. [28] reported that ANuA was positive in 61 of 95 (64%) patients with SLE, none of 95 healthy controls, none of 28 fibromyalgia patients, and in only 2 of 48 (4%) rheumatoid arthritis (RA) controls. In a study of 131 SLE patients by Düzgün et al. [6], 72 (54.9%) were seropositive for ANuA, which was significantly higher than only 3 of 74 (4%) patients with RA while none of the patients with scleroderma and 50 healthy individuals were seropositive. The sensitivity and specificity of ANuA in SLE were 83.6% and 70%, respectively. 38.9% of these SLE patients had renal involvement. Among these patients, ANuA positivity and anti-dsDNA were 74.5% and 78.4%, respectively, and ANuA were found to be 31.4% positive in SLE patients lacking anti-dsDNA antibody. In a Korean study, 98 of 129 patients (76%) with SLE presented ANuA, and 15 (60%) patients had ANuA among the 25 patients without anti-dsDNA antibodies [26].

At the clinical interface, ANuA are predominantly associated with renal involvement [26–29, 57, 85] although hematological involvement or arthritis, malar rash, pleuritis, and oral ulcers have also been observed [85]. Another study reported that the frequency of ANuA in patients with active SLE is similar to that for anti-dsDNA antibodies [27, 35]. Of note, ANuA are positively correlated with anti-ds-DNA antibodies titers [26, 28, 29, 93]. The clinical value and serological independence of ANuA was highlighted by data showing that 11% to 51% of ANuA positive sera did not have anti-dsDNA antibodies [26, 28, 57, 94, 96]. Amoura et al. [27] showed a correlation between ANuA and disease activity only for the IgG3, as well as for the IgG/IgM ratio, taking into consideration that IgM-class anti-dsDNA antibodies seem to have a protective role in autoimmune nephropathy [12, 99]. In a more recent study by Villalta et al. [100] 200 SLE patients with glomerulonephritis showed significantly elevated levels of IgA anti-dsDNA, anti-dsDNA IgG/IgM, and IgA/IgM ratios than patients without renal disease. In a report by Souza et al. [101], ANuA were more prevalent in active SLE patients (74.2%) than in inactive SLE (11.7%). In this study, ANuA also correlated with disease activity and renal damage but were also found in a proportion of sera that did not have anti-dsDNA antibodies. They also observed that ANuA were not found in cutaneous lupus erythematosus but were helpful in supporting the diagnosis of DIL, especially lupus related to procainamide, quinidine, and hydralazine exposure. Andreoli et al. [102] studied a cohort of 105 patients with primary anti-phospholipid antibody syndrome (PAPS) of which 77% were positive for ANuA, whereas medium-high titres were only detected in 46%. They concluded that ANuA were more frequently detected in PAPS and lupus like disease, although no relationship with clinical/serological features was found, except for a weak correlation with anti-dsDNA antibodies. An intriguing report by Bossuyt et al. [103] indicted that ANuA in the absence of anti-dsDNA can be used as a serological discriminator to identify patients with TNP-alpha inhibitor related ANA.

Some studies have explored the presence of ANuA in the context of relevant circulating autoantigens. For example, significantly higher levels of nucleosomes were found in plasma of 13/58 (22.4%) SLE patients as compared to healthy individuals [104]. Similar results were obtained using a highly sensitive Picogreen assay (Life Technologies), to quantify DNA in sera of SLE patients [105]. Curiously, none of the 13 patients with elevated levels of circulating DNA as detected by Picogreen had detectable ANuA or anti-dsDNA antibodies. Interestingly, Amoura et al. [104] demonstrated that there was an inverse correlation between DNA concentration, ANuA, and anti-dsDNA antibodies. Derksen et al. [77] measured DNA levels in circulating immune complexes by a quantitative immunochemical assay and found a decrease in DNA concentration during severe flares of SLE and an inverse correlation between DNA levels in immune complexes and anti-dsDNA antibody concentrations measured by CLIFT, ELISA, plasmid DNA-based assay, and Farr assay. A similar inverse relationship between DNA and anti-dsDNA antibodies in plasma from SLE patients was also observed by McCoubrey-Hoyer et al. [106], although many patients had high levels of plasma DNA and anti-dsDNA antibodies without clinical nephritis. This suggests that factors other than simply the presence of ANuA and/or anti-dsDNA antibodies might be important in the initiation and perpetuation of glomerular damage in SLE. In another study, Jørgensen et al. [107] demonstrated an inverse correlation between anti-dsDNA antibodies and the DNA concentration (measured by quantitative PCR) in the circulation in both murine and human serum samples of SLE. High titer of anti-DNA antibodies in human sera correlated with reduced levels of circulating chromatin and in lupus prone mice with deposition within glomeruli. The inverse correlation between DNA concentration and anti-dsDNA antibodies perhaps reflected antibody-dependent deposition of immune complexes during the development of lupus nephritis in autoimmune lupus prone mice. Williams et al. [108] demonstrated that the levels of circulating nucleosomes were raised in SLE patients with active central nervous system disease and renal involvement. However, this is in contrast to previous reports from Derksen et al. showing decreased levels of circulating nucleosomes during flares of the disease [77].

## 7. Juvenile SLE (JSLE)

In children, anti-dsDNA antibody testing was slightly more sensitive than ANuA (66.5% versus 64.1%), while specificity slightly favored ANuA (98.8% versus 97.1%). However, ANuA displayed a more relevant predictive value than anti-dsDNA antibodies (reviewed in [8]). In a study by Keusseyan et al. [109], anti-nucleosome core particle and anti-chromatin antibodies exhibited high specificity for JSLE but had a similar frequency in active and inactive disease. They also observed that anti-chromatin and IgG/IgG3 anti-nucleosome core particle serum levels did not differ between active and inactive JSLE. Disease activity correlated with anti-dsDNA antibodies but not with antibodies to other chromatin components. Notably, anti-nucleosome antibodies in the absence of anti-dsDNA were observed in 14% of their patients.

Campos et al. [110] studied ANuA and anti-dsDNA by ELISA in 74 patients with JSLE and 64 normal controls. The presence of ANuA was significantly associated with higher SLEDAI scores, malar erythema, hemolytic anemia, anti-dsDNA, and low complement levels, but not with renal manifestations. In a cohort of 67 JSLE patients, Jesus et al. [111] observed a higher frequency of ANuA and anti-dsDNA antibodies (48% and 69%, resp.) as compared to controls although anti-dsDNA was associated with higher disease activity scores. Wu et al. [112] compared the serum levels of ANuA in 30 JSLE patients by ELISA to 29 adult SLE patients, 30 healthy controls, 21 juvenile idiopathic arthritis, and 23 Henoch-Schonlein purpura patients as autoimmune disease controls. The mean ANuA titer in the JSLE patients was higher than those of adult SLE patients, normal and disease controls. The prevalence of both ANuA (90%) and anti-dsDNA (76.7%) in JSLE patients was higher than that in adult SLE patients (58.6% and 48.3%). A positive correlation was demonstrated between ANuA and anti-dsDNA as well as the SLEDAI scores and an inverse correlation with C3 complement in pediatric and adult patients.

## 8. Future Areas of Interest

A number of more recent observations have significant importance in the field of anti-chromatin antibodies. This includes a reinvigorated study of HMG proteins triggered by newer observations on the role of HMGB1 in inflammation and the intriguing evidence that the formation of DNA extracellular traps (NETs) and extracellular microvesicles may provide other vehicles by which inflammatory and immunogenic components of chromatin can be released into the extracellular environment. Although not discussed in detail here, recent evidence suggests that the use of nucleosomal peptides as tolerogens may be a successful approach to suppressing certain aspects of lupus pathogenesis and could lead to the design of novel therapeutics [113].

### 8.1. High Mobility Group Proteins

High mobility group (HMG) proteins are operationally defined by their extractability from chromatin in 0.35 M NaCl and their solubility in 5% perchloric acid (PCA) and 2% trichloroacetic acid (TCA) [114, 115]. As potential autoantigens, HMG's are interesting proteins because they are highly amphipathic, with both basic and acidic domains, and they have a relatively high content of proline [114, 115]. The five main families of mammalian HMG proteins are designated HMGA, HMGB, HMGN, and SOX and TYCF transcription factors (Table 2) [116]. Each of these proteins has been shown to have unique chromatin-binding motifs and characteristics. For example, HMGA contains an AT hook; HMGB contains a HMG box domain; HMGN (including HMG-14/HMGN1 and HMG-17/HMGN2) contains a nucleosomal binding domain. There is evidence that HMG-14 and HMG-17 are preferentially associated with transcriptionally active chromatin [117, 118]. HMGN1/HMG-14 and HMGN2/HMG-17 bind to nucleosomal core histones of transcriptionally active chromatin [119, 120]. In comparison, HMGB1/HMG-1 and HMGB2/HMG-2 are associated with internucleosomal DNA and appear to be evenly distributed in active and nonactive genes [115, 121].

Dating to the seminal studies of Bustin et al. in 1982 [122], HMG antibodies have been reported in SLE [122–124], mixed connective tissue disease [122], juvenile idiopathic arthritis (JIA) [125–128], canine lupus [129], DIL [130], systemic sclerosis [131], other systemic autoimmune rheumatic diseases [132], primary pulmonary hypertension [133], inflammatory bowel disease [134–136], primary biliary cirrhosis [137], type I diabetes [138], autoimmune hepatitis [139], septic shock [140], and liver transplant patients (see Table 2 for more details) [141, 142].

In a study of sera from 42 DIA patients, we found reactivity with HMG proteins 14 and/or 17 in 67% of the sera by immunoblotting assays and in 58% by an ELISA [130]. The slightly lower percentage in the ELISA may be explained in part by the high cut-off value (mean + 3SD) for the ELISA. Some difference in binding might also be expected because of a different conformation and orientation of proteins on the solid matrix of the two assays. Nevertheless, there was correlation between high absorbance values obtained by ELISA and strongly positive immunoblots. By comparison, reactivity with HMG-1 and/or HMG-2 was observed in only 9/42 (21%) of the DIA sera, although anti-HMG antibodies were found in patients from each drug group with the exception of a serum from a patient treated with alpha methyldopa [130]. Because the prevalence of anti-HMG antibodies in symptomatic DIL was similar to that in asymptomatic PA- or INH-treated patients, anti-HMG antibodies do not appear to correlate with disease manifestations. Other studies reported autoantibodies to HMG-17 in 10/29 (34%) SLE sera [122]and in 47% of pauciarticular onset JRA [126] and antibodies to HMG-1 and HMG-2 in 10% of SLE [122] and 39% of JRA patients [125]. Another report found that 6% and 18% of sera from canine lupus reacted with HMG-1 and HMG-2, respectively, but no antibodies bound to HMG-14 and HMG-17 [129].

Until the last decade, one of the HMG proteins, HMGB1, was primarily regarded as a DNA-binding protein that participated in chromatin structure and transcriptional regulation [143, 144]. However, HMGB1 gained particular interest in the last decade after it was shown that it had a proinflammatory role in endotoxin lethality in mice and in sepsis [145] after its release from damaged or necrotic cells [146, 147]. HMGB1 is a ubiquitous and abundant chromatin component, and it is currently well known as one of the damage-associated molecular pattern molecules (DAMPs) interacting with the receptor for advanced glycation end product (RAGE), toll-like receptor (TLR)2, TLR4, and TLR9 (reviewed in [146, 148, 149]). The proinflammatory roles of HMGB1 have been reported in acute lung inflammation [150], atherosclerosis, and restenosis after vascular damage [151], hepatic injury after murine liver ischemia reperfusion [152], acute pancreatitis [153], rheumatoid arthritis [154], pulmonary fibrosis [155], cerebral ischemia [156], Kawasaki disease [157], cold ischemia/reperfusion-induced inflammation [158], acute appendicitis [159], systemic inflammatory response syndrome [160, 161], febrile seizures [162], hyperlipidemia [163], preeclampsia [164], and models of liver failure [165–167].

HMGB1 is also secreted from various cell types during activation and/or cell death and may act as a proinflammatory mediator, alone or as part of DNA-containing immune complexes in SLE [168, 169]. A recent study by Wen et al. [170] concluded that HMGB1 in circulating DNA-containing immune complexes was crucial for anti-dsDNA Ab induction and it correlated positively with anti-dsDNA Ab production in patients with SLE. They also observed that TLR2/MyD88/microRNA-155 (miR-155) pathway was pivotal for HMGB1 to confer anti-dsDNA Ab induction. Recent studies evaluating the role of HMGB1 in LN showed that 21/69 SLE patients with biopsy proven active LN had higher urinary and serum levels of HMGB1 as compared to those without active LN [171]. They also concluded that the serum levels of HMGB1 correlated with SLE disease activity score and, inversely, with levels of the complement components C3 and C4 [171]. Similarly, a study of 35 active SLE LN patients showed that renal tissue expression and serum levels of HMGB1 were increased in LN and HMGB1 failed to decrease in serum and tissue after immunosuppressive therapy, a feature reflecting persistent inflammatory activity [171]. Santoro et al. [172] revealed a high prevalence of autoantibodies to HMG protein structure specific recognition protein I (SSRP1) in 28.8% of patients with SLE as compared to other autoimmune disorders. In a study by Li et al. [173] HMGB1 correlated with disease activity, low complements, and disrupted cytokine homeostasis. In cutaneous lupus, increased amounts of cytoplasmic and extracellular HMGB1 were detected within the lesional skin together with high expression levels of TNFa and IL-1b [8]. Future research on HMG proteins promises to yield other related novel biomarkers [154, 157, 162, 164] and therapeutic targets in SLE [153, 165, 174–178].

Last, a few reports have implicated autoantibodies to sex related Y HMG box (SOX) proteins in type I diabetes [138] and anti-SOX13 in primary biliary cirrhosis, autoimmune hepatitis, and other diseases [137]. In this latter study, anti-SOX13 was detected in 18% of patients with PBC, 13% with autoimmune hepatitis, and 18% with type 1 diabetes, at lower frequencies in other conditions including the multisystem autoimmune diseases, SLE, and rheumatoid arthritis, but in only 1% of normal sera. More studies are required to validate these studies and determine their sensitivity and specificity.

Observations that there is a high frequency of antibodies to the HMG proteins associated with nucleosomes add further evidence implicating the nucleosome or nucleosomal subunits as immunogens. Furthermore, observations that the most common autoantibody targets in human diseases are HMGN1/HMG-14 and HMGN2/HMG-17, which are preferentially associated with the nucleosomes of transcriptionally active chromatin, suggests that functionally and structurally distinct forms of nucleosomes are the inciting immunogens in autoimmune disease.

### 8.2. DNA Neutrophil Extracellular Traps (Nets)

Neutrophils kill certain extracellular pathogens by releasing their highly decondensed chromatin as extracellular traps (NETs) [179]. The impact of NETs is derived from the combined antimicrobial activities of granular components, histones, and some cytoplasmic proteins (reviewed in [180, 181]) and the release of danger signals or DAMPs from disrupted cells and tissues [182]. The various methods of NET quantification include microscopy [179, 183–185] and DNA detection either with membrane impermeable DNA dyes [179] or by staining the DNA in the supernatant after releasing the NETs with a mild nuclease treatment [181, 186]. The role of NETs in autoimmune diseases has been the subject of recent reviews [187, 188]. Neutrophils isolated from SLE patients are more prone to making NETs, particularly in response to antibody complexes [189–192]. As discussed throughout this review, SLE is characterized by autoantibodies directed against DNA, chromatin, and DNA-associated proteins, all potential components of NETs. Recent evidence points to an imbalance between NET formation and NET clearance in SLE [185, 190–192] and decreased NET degradation has been associated with complement activation [193] and correlated with a subset of SLE patients with renal disease and attended by DNase1 inhibitors and anti-NET antibodies [185]. There is also preliminary evidence that the nuclear material externalized in NETs has antiviral activity [194, 195] and is involved in vasculopathies [196, 197], sterile inflammation [198], and promote autoantibody formation in SLE [191]. However, further studies are needed to understand the complex role of these NETs in triggering an autoimmune response and/or inflammation that would aid in predicting disease onset or flares and facilitate the development of compounds that selectively target the deleterious aspects triggered by these macromolecular lattices.

## 9. Summary

1. ANuA and anti-dsDNA are independent and complementary biomarkers that have value in the diagnosis and disease activity assessment of SLE. ANuA are specially a useful marker in the diagnosis of SLE patients who are anti-dsDNA negative. ANuA and anti-dsDNA both are associated with disease flare and lupus nephritis.
2. In general, ANuA have equal specificity but higher sensitivity, positive likelihood ratio, and diagnostic odds ratio than anti-dsDNA antibodies for the diagnosis of SLE.
3. The emergence of newer immunoassays for the detection of antibodies to chromatin components requires ongoing pre- and postmarketing evaluation. Standardization of nomenclature and assay performance is a desirable goal.
4. The renaissance of autoantibodies to HMG proteins and their relationship to other chromatin components including nucleosomes and DNA NETs are beginning to weave interesting paradigms in autoimmunity that requires further investigation. Circulating anti-HMGB1 and HMGB1 levels may be useful when assessing the protective effects of autoantibodies.

## Figures and Tables

**Table 1:** ANuA in SLE compared to controls*.

Authors [reference]	ANuA in SLE cohort sensitivity/specificity (*n*)	% anti-dsDNA in SLE cohort	ANuA clinical and serological associations
Sardeto et al., 2012 [88]	62/100 (92)	40	Disease activity, anti-dsDNA
Suleiman et al., 2009 [89]	52/98 (90)	37	Disease activity, anti-DNA
Kim et al., 2008 [95]	98/78 (100)	nr	Leucopenia, low complement, disease activity, anti-dsDNA
Bossuyt et al., 2008 [103]	78/73 (40)	65	Proteinuria, disease flare, ANuA but not anti-dsDNA found in TNF alpha inhibitor related ANA
Grootscholten et al., 2007 [83]	81/nr (52) (LSLN)	96	Disease activity but not disease course, LN, anti-histone, anti-C1q
Su et al., 2007 [96]	62/98 (233)	nr	Disease activity, malar rash, arthralgia, anti-dsDNA
Düzgün et al., 2007 [6]	55/98 (131)	nr	Disease activity, LN, anti-dsDNA
Tikly et al., 2007 [97]	45/94 (86)	nr	Disease activity, discoid lupus/malar rash, anti-dsDNA, aCL
Braun et al., 2007 [94]	64/99 (78)		Disease activity, anti-dsDNA, anti-C1q
Campos et al., 2006 [110]	53/98 (74) (JSLE)	54	Disease activity, malar rash, hemolytic anemia, low complement, anti-dsDNA
Simón et al., 2004 [85]	100/97 (73)	63	LN, malar rash, arthritis, oral ulcers, pleuritis, disease activity, renal damage
Ghirardello et al., 2004 [93]	86/95 (101)	69	Hematological, anti-dsDNA
Cairns et al., 2003 [28]	64/99 (95)	nr	LN, hematological, anti-dsDNA
Cervera et al., 2003 [29]	69/92 (100)	nr	LN, anti-dsDNA
Min et al., 2002 [26]	76/98 (129)	79	Disease activity, low complement, anti-dsDNA
Amoura et al., 2000 [27]	72/90 (120)	nr	Disease activity, LN

aCL: anticardiolipin; CLE: cutaneous lupus erythematosus, ANuA: anti-nucleosome antibodies; dsDNA: double-stranded DNA; JSLE: juvenile systemic lupus erythematosus; LN: lupus nephritis; nr: not reported; NRL: nonrenal lupus; LSLN: longitudinal study of lupus nephritis; nr: not reported.

*Some data adapted from [4, 8].

**Table 2:** High mobility group proteins (HMG): classification, nomenclature, and autoimmunity.

HMG family members	Functional domain	Autoantibodies	Other immune reactions
HMGA HMGA1 HMGA2	AT hook Modulate transcription by altering chromatin architecture		

HMGB HMGB1/HMG1* HMGB2/HMG2* HMGB3 HMGB4	HMG box domain Bind minor groove of DNA in a nonsequence-dependent fashion	AIH, SLE, SSc, DIA, JIA, UC, septic shock, liver transplant patients	HMGB1: Proinflammatory mediator. Perhaps anti-HMGB1 autoantibodies ameliorate proinflammatory effects and are protective

HMGN HMGN1/HMG14* HMGN2/HMG17*	HMG nucleosomal binding domain Function in transcription, replication, DNA repair, alter the interaction of histone H1 with nucleosomes to maintain a decondensed chromatin structure	DIA/DIL, SSc, PPH, SLE, MCTD	

SOX SDRY SOXB1 SOXB2 SOX-C, -D, -E, -F SOX13	Transcription Factors HMG box with high sequence similarity to SDRY	PBC, AIH, type I diabetes	

TCF LEBF1 TCTF1 TF7L1P TF7L2P	Transcription factors	None published	Role in control of T cell transcription

*Older nomenclature HMG1, HMG2, HMG14, HMG17.

AIH: autoimmune hepatitis; DIA: drug-induced autoimmunity, DIL: drug-induced lupus; JIA: juvenile idiopathic arthritis; LEBF: lymphoid enhancer-binding factor 1, MCTD: mixed connective tissue disease; PBC: primary biliary cirrhosis; PPH: primary pulmonary hypertension; SARD: systemic autoimmune rheumatic diseases; SDRY: sex determining region Y; SLE: systemic lupus erythematosus; SOX: Sry-related HMG box; SSc: systemic sclerosis; TCF: transcription factor; TCTF: T cell transcription factor; TF7LP: Transcription factor 7-like Protein; UC: ulcerative colitis.

## Abbreviations

ALBIA: Addressable laser bead immunoassays
ANuA: Anti-nucleosome autoantibodies
CLIFT: Crithidia luciliae immunofluorescence test
DAMPS: Damage-associated molecular patterns
dsDNA: Double stranded DNA
EliA: Fluoroenzyme immunoassay
ELISA: Enzyme-linked immune assays
HMG: High mobility group
JIA: Juvenile inflammatory arthritis
JSLE: Juvenile systemic lupus erythematosus
MCTD: Mixed connective tissue disease
NETs: Neutrophil extracellular traps
PAPS: Primary anti-phospholipid antibody syndrome
RA: Rheumatoid arthritis
SLE: Systemic lupus erythematosus
SOX: Sex related Y HMG box
ssDNA: Single stranded DNA.
